# Angiotensin-Converting Enzyme Insertion/Deletion Polymorphism Is Not a Major Determining Factor in the Development of Sporadic Alzheimer Disease: Evidence from an Updated Meta-Analysis

**DOI:** 10.1371/journal.pone.0111406

**Published:** 2014-10-31

**Authors:** Xue-bin Wang, Ning-hua Cui, Jie Yang, Xue-ping Qiu, Jia-jia Gao, Na Yang, Fang Zheng

**Affiliations:** 1 Center for Gene Diagnosis, Zhongnan Hospital of Wuhan University, Wuhan, Hubei, China; 2 Department of Clinical Laboratory, Children's Hospital of Zhengzhou, Zhengzhou, Henan, China; Max-Delbrück Center for Molecular Medicine (MDC), Germany

## Abstract

Angiotensin-converting enzyme gene (*ACE*) insertion/deletion (I/D) polymorphism have long been linked to sporadic Alzheimer disease (SAD), but the established data remained controversial. To clarify this inconsistency, a comprehensive meta-analysis was conducted. Through searching of Pubmed, Embase, Alzgene, China National Knowledge Infrastructure (CNKI) and manually searching relevant references, 53 independent studies from 48 articles were included, involving a total of 8153 cases and 14932 controls. The strength of association was assessed by using odds ratios (ORs) with 95% confidence intervals (CIs). Further stratified analyses and heterogeneity analyses were tested, as was publication bias. Overall, significant associations were revealed between I/D polymorphism and SAD risk using allelic comparison (OR = 1.09, 95%CI = 1.01–1.17, p = 0.030), homozygote comparison (OR = 1.17, 95%CI = 1.01–1.34, p = 0.030) and the dominant model (OR = 1.16, 95%CI = 1.04–1.29, p = 0.008), but they were not sufficiently robust to withstand the false-positive report probability (FPRP) analyses. Otherwise, in subgroup analyses restricted to the high quality studies, the large sample size studies and studies with population-based controls, no significant association was observed in any genetic models. In summary, the current meta-analysis suggested that the *ACE* I/D polymorphism is unlikely to be a major determining factor in the development of SAD.

## Introduction

Alzheimer's disease (AD) is the most common form of dementia in the elderly, and it is characterized by progressive memory loss and cognitive dysfunction [1]. Although some AD cases are familial, about 90% are sporadic [2]. Sporadic Alzheimer's disease (SAD) is considered to be a multifactorial disease with a complex interaction of both genetic and environmental factors [3]. One of the proposed mechanisms for SAD is the amyloid hypothesis, which suggests that deposition of beta-amyloid (Aβ) is a primary event in the pathological cascade for SAD [4]. The balance between the expression and the degradation of Aβ changes, and aggregate of Aβ would cause complex reactions, such as phosphorylation of protein Tau [5], loss of neurotransmitter [6] and finally formation of senile plaques (SP), as well as intracellular neurofibrillary tangles.

Recently, growing evidence has implicated angiotensin-converting enzyme (*ACE*), a zinc metalloprotease widely expressed in brain, as a possible modulator of Aβ metabolism [7]. The *ACE* gene is located on chromosome 17q23 and consists of 26 exons and 25 introns. The most common polymorphism of *ACE* gene is the insertion/deletion (I/D) variant of 287 base pairs in intron 16, which has been suggested to be associated with serum *ACE* protein levels [8], the specific activity of *ACE* protein domain [9], the transcriptional activity of *ACE* gene promoter region [10] and resulted in the susceptibility to SAD. A study published in 1999 first reported the association between the I/D polymorphism and SAD in a combined sample of three case-control samples from the United Kingdom [11]. Since then, a great number of studies have been performed on this polymorphism with SAD risk in different populations but have generated equivocal results. Therefore, in 2003–2005, three meta-analyses [12]–[14] have been published and implied possible association of the I/D polymorphism on AD risk. However, there were more and further single studies after 2005. Hence, an updated meta-analysis combining all available studies was performed to derive a more precise estimation of this relationship.

## Materials and Methods

### Literature search

This meta-analysis was performed according to the methodology advocated by the PRISMA statement [15]. All studies included in the meta-analysis were selected by searching the Pubmed, EMBASE, Alzgene and China National Knowledge Infrastructure (CNKI) databases up to May 2014 using the following keywords: “(Alzheimer or AD) and (angiotensin-converting enzyme gene or *ACE*) and (polymorphism or variant or genotype)”. In addition, the reference lists of reviews and retrieved articles were checked for potential studies. Articles that reported results from more than 1 population were considered as separate studies. Only studies published in English or Chinese were included.

### Inclusion criteria

The studies included in the meta-analysis were required to meet the following criteria: (1) case-control or cohort design; (2) association between I/D polymorphism and SAD risk; (3) application of standardized clinical or pathologic criteria for the diagnosis of SAD; (4) sufficient genotype distributions for calculation of odds ratios (ORs) with 95% confidence intervals (CIs);

The following were excluded: (1) reports with incomplete data; (2) review articles, abstracts, case reports; (3) studies based on familiar Alzheimer's disease (FAD) or mild cognitive impairment (MCI); (4) studies about other *ACE* polymorphisms. When the articles contained duplicated data, the most recent or complete studies were selected.

### Data extraction

Data were extracted from eligible articles independently by two of the authors, with any disagreement resolved by consensus. The following information was collected in a predefined data collection form: first author's name, publication year, country, geographical location of participants (North European, South Caucasian, Asian, etc), sample size, AD diagnosis criteria, genotyping methods, source of controls, risk allele frequency in controls, results of Hardy-Weinberg equilibrium (HWE) in controls and quality assessment of studies.

### Quality score assessment

The quality of each study was independently assessed by two authors who used quality scoring criteria modified from previous studies [16], [17] (Table S1 in File S1). The modified criteria cover the representativeness of cases, the credibility of controls, genotyping examination, association assessment and total sample size. Quality scores ranged from 0 point (worst) to 12 points (best). Studies scoring higher than 9 points were classified as high quality.

### Statistical analysis

First, deviance from HWE was assessed for the controls of each study using the chi-squared test**.** Second, genotype distributions of controls were used to estimate the frequency of the putative risk allele (I allele) in various geographic location using the inverse variance method [16]. Third, we mainly examined the overall effects for I/D polymorphism. Briefly, the pooled ORs along with their corresponding 95% CIs were estimated for allelic comparison (I vs D), additive model (homozygote comparison: II vs DD; heterozygote comparison: ID vs DD), the recessive model (II vs ID+DD) and the dominant model (II+ID vs DD).

Cochran's *Q* statistic was used to test for heterogeneity, and the percentage variability of the heterogeneity between studies was quantified using the *I^2^* statistic. Thus, *I^2^* values around 25%, 50% and 75% would indicate low, medium and high heterogeneity respectively [18] **.** The random-effect model (DerSimonian-Laird) [19] was used to assess pooled ORs when *I^2^* (%)>50% or *P (Q)*<0.10. Otherwise, the fixed-effect model (Mantel-Haenszel) [20] was used. Subgroup analyses were performed, when feasible, according to geographic location (ethnicity), sample size, quality appraisal score, genotyping methods, source of controls, and publication time. In addition, a meta-regression procedure was adopted to find potential sources of heterogeneity [21]. Further, Galbraith plots were used to visualize the impact of individual studies on the overall homogeneity, which identified the outliers as possible major sources of heterogeneity [22]. Cumulative meta-analyses of associations for I/D polymorphism were also performed to investigate the trend and the stability of risk effects as evidence accumulated over time (by publication year). In addition, sensitivity analyses were carried out to evaluate the stability of the results through sequential removal of each study or after excluding those studies that deviated from HWE. Moreover, sensitivity analyses limited to only the English-language studies were conducted to investigate the influence of Chinese-language studies on the overall meta-analysis. Publication bias was assessed graphically by funnel plots [23] and formally by Egger's tests [24] and Begg’s tests [25], given a significant value of 0.05. All of the above analyses were conducted using RevMan 5.2 (The Nordic Cochrane Centre, The Cochrane Collaboration) & STATA 12.0 (Stata, College, TX, USA).

For each statistically significant association, the false-positive report probability (FPRP) analyses were performed using the method reported by Wacholder et al [26]. The FPRP value is determined by the p value, the prior probability for the association, and statistical power. We calculated FPRP assuming a prior probability of 0.001 as previously proposed [27] for candidate gene analyses. Statistical power was based on the ability to detect an OR of 1.5, with α equal to the observed p value. An FPRP cutoff value of 0.2 was used [26] and only the results with FPRP value less than 0.2 were referred as noteworthy. Statistical power and FPRP analyses were computed using the Excel spreadsheet provided by Wacholder et al [26].

## Result

### Study characteristics

**Figure 1:**
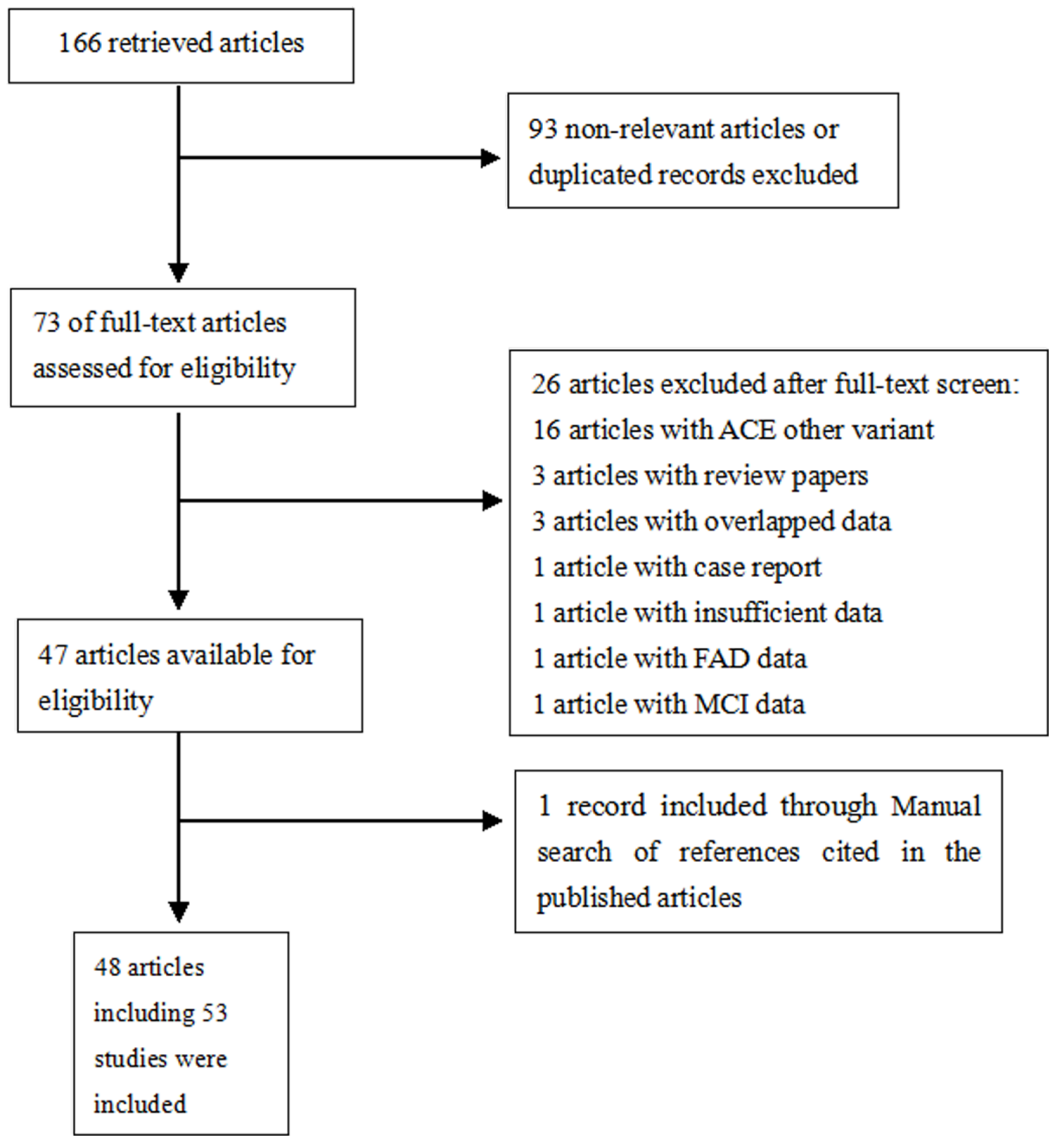
Flow chart of article selection in our meta-analysis.

A total of 166 relevant articles were initially identified from Pubmed, EMBASE, Alzgene and CNKI databases. After titles and abstracts were screened, 93 articles were excluded because of irrelevant data. The full texts of the remaining 73 records were carefully reviewed (Fig. 1). Among these articles, 16 articles were about other *ACE* SNPs; two articles were excluded because of FAD [28] or MCI data [29]; four articles were excluded due to case reports [30] or review papers [31]–[33]; four articles were excluded owing to overlapped [34]–[36] or insufficient data [37]. Manual search of references cited in the published articles revealed one additional article [38]. Four of the eligible articles [11], [39]–[41] contained data from 9 independent studies. Therefore, 48 articles including 53 studies were included in the present meta-analysis [11], [12], [14], [38]–[82]. Among these studies, a total of 18 studies reported on North European populations; 14 studies on South Caucasians (defined here as from the Mediterranean or Jews); 15 studies on Asians and 6 studies on other ethnicities. Deviation of HWE was detected in the control subjects of eight studies [39], [40], [48], [50], [52], [64], [66], [71]. The flowchart for the process of including/excluding articles is shown in Figure 1, and the characteristics of all included studies are summarized in Table 1.

**Table 1:** Characteristics of included studies in this meta-analysis evaluating the effects of I/D polymorphism on the risk of developing SAD.

No.	First author (Year)	Country	Ethnicity	Sample size (Case/Control)	Criteria for SAD diagnosis	Genotyping method	Source of control	I allele frequency (%)	HWE (p)	Quality score
1	Scacchi 1998 [42]	Italy	South Caucasian	80/153	NINCDS/ADRDA	PCR (original primer)	P	36.9	Y (0.764)	8
2	Chapman 1998 [43]	Israel	South Caucasian	49/40	NINCDS/ADRDA and DSM-III-R	PCR (original primer)	P	33.8	Y (0.754)	7
3	Kehoe (London) 1999 [11]	UK	North European	135/111	NINCDS/ADRDA	PCR (insertion specific primer)	P	41.4	Y (0.251)	9
4	Kehoe (Belfast) 1999 [11]	UK	North European	209/198	NINCDS/ADRDA	PCR (insertion specific primer)	P	51.5	Y (0.485)	10
5	Kehoe (Cardiff) 1999 [11]	UK	North European	198/77	NINCDS/ADRDA	PCR (insertion specific primer)	P	40.3	Y (0.820)	10
6	Palumbo 1999 [44]	Italy	South Caucasian	140/40	NINCDS/ADRDA	PCR (insertion specific primer)	P	37.5	Y (0.109)	9
7	Hu 1999 [45]	Japan	Asian	132/148	NINCDS/ADRDA	PCR (original primer)	P	61.8	Y (0.618)	10
8	Alvarez 1999 [46]	Spain	South Caucasian	350/517	NINCDS/ADRDA	PCR (insertion specific primer)	P	37.3	Y (0.458)	11
9	Farrer (Moscow) 2000 [39]	Moscow	North European	151/206	NINCDS/ADRDA	PCR (original primer)	M	47.6	Y (0.221)	11
10	Farrer (Mixed) 2000 [39]	Mixed	North American	235/162	NINCDS/ADRDA	PCR (original primer)	P	38.6	N (0.022)	10
11	Mattila 2000 [47]	Finland	North European	80/67	NINCDS/ADRDA and CERAD	PCR (insertion specific primer)	P	38.1	Y (0.879)	9
12	Crawford 2000 [48]	USA	North American	171/175	NINCDS/ADRDA	PCR (insertion specific primer)	P	38.3	N (0.018)	11
13	Myllykangas 2000 [49]	Finland	North European	121/75	NINCDS/ADRDA and CERAD	PCR (original primer)	A	57.3	Y (0.166)	9
14	Yang 2000 [50]	China	Asian	188/227	NINCDS/ADRDA and DSM-IV	PCR (original primer)	H	57.5	N (<0.001)	8
15	Narain 2000 [52]	UK	North European	239/342	CERAD	PCR (insertion specific primer)	P	47.2	N (<0.001)	10
16	Isbir 2000 [51]	Turkey	South Caucasian	35/29	NINCDS/ADRDA	PCR (original primer)	P	48.3	Y (0.356)	7
17	Zuliani 2001 [53]	Italy	South Caucasian	40/54	NINCDS/ADRDA	PCR (original primer)	P	42.6	Y (0.317)	9
18	Perry 2001 [54]	USA	African American	111/78	NINCDS/ADRDA	PCR (original primer)	P	32.1	Y (0.302)	9
19	Richard (Cohort 1) 2001 [40]	France	South Caucasian	433/475	NINCDS/ADRDA and DSM-III-R	PCR (insertion specific primer)	P	45.2	Y (0.833)	11
20	Richard (Cohort 2) 2001 [40]	France	South Caucasian	56/221	NINCDS/ADRDA and DSM-III-R	PCR (insertion specific primer)	M	50.7	N (0.050)	10
21	Buss 2002 [55]	Europe	North European	261/306	NINCDS/ADRDA	PCR (insertion specific primer)	P	46.7	Y (0.618)	10
22	Wu 2002 [56] a	China	Asian	96/96	DSM-IV	PCR (original primer)	P	49.5	Y (0.839)	9
23	Lendon 2002 [57]	UK	North European	214/99	NINCDS/ADRDA and DSM-III-R	PCR (original primer)	P	49.5	Y (0.365)	10
24	Monastero 2002 [58]	Italy	South Caucasian	149/149	NINCDS/ADRDA	PCR (original primer)	P	39.9	Y (0.444)	10
25	Cheng 2002 [59]	China	Asian	173/116	NINCDS/ADRDA	PCR (insertion specific primer)	P	64.7	Y (0.545)	11
26	Panza 2003 [60]	Italy	South Caucasian	141/268	NINCDS/ADRDA	PCR (insertion specific primer)	P	37.7	Y (0.115)	11
27	Seripa (Italy) 2003 [41]	Italy	South Caucasian	126/106	NINCDS/ADRDA	PCR (insertion specific primer)	P	37.7	Y (0.708)	10
28	Seripa (USA) 2003 [41]	USA	North American	124/97	NINCDS/ADRDA and CERAD	PCR (insertion specific primer)	A	45.4	Y (0.098)	10
29	Kehoe 2003 [12]	UK	North European	333/109	CERAD	PCR (insertion specific primer)	A	45.9	Y (0.681)	11
30	Carbonell 2003 [61]	UK	North European	80/65	NINCDS/ADRDA	PCR (original primer)	M	49.2	Y (0.903)	8
31	Camelo 2004 [62]	Columbia	South American	83/68	NINCDS/ADRDA	PCR (original primer)	P	47.8	Y (0.456)	7
32	Chalmers 2004 [63]	UK	North European	83/58	CERAD	PCR (insertion specific primer)	A	43.1	Y (0.904)	9
33	Feng 2004 [64] a	China	Asian	26/68	DSM-IV	PCR (insertion specific primer)	P	60.3	N (<0.001)	7
34	Lehmann 2005 [14]	UK	North European	203/248	NINCDS/ADRDA	PCR (original primer)	M	46.2	Y (0.713)	10
35	Kolsch 2005 [65]	Germany	North European	351/348	DSM-IV	PCR (insertion specific primer)	P	45.3	Y (0.174)	12
36	Zhang 2005 [66]	China	Asian	192/195	NINCDS/ADRDA and DSM-IV	PCR (original primer)	P	65.4	N (0.006)	8
37	Sleeger 2005 [67]	Netherland	North European	250/6403	DSM-III-R	PCR (insertion specific primer)	P	47.1	Y (0.428)	12
38	Keikhaee 2006 [68]	Iran	Asian	117/125	NINCDS/ADRDA	PCR (original primer)	P	43.2	Y (0.806)	8
39	Meng 2006 [69]	Israel	South Caucasian	92/166	NINCDS/ADRDA	PCR (original primer)	P	31.0	Y (0.993)	8
40	Wehr 2006 [70]	Poland	North European	100/144	NINCDS/ADRDA	PCR (insertion specific primer)	P	48.6	Y (0.492)	9
41	Wang B 2006 [71]	China	Asian	104/128	DSM-III-R	PCR (original primer)	P	62.1	N (0.013)	7
42	Wang HK 2006 [72]	China	Asian	151/161	NINCDS/ADRDA and DSM-IV	PCR (insertion specific primer)	P	76.1	Y (0.929)	11
43	Nacimas 2007 [73]	Italy	South Caucasian	235/303	DSM-IV	PCR (insertion specific primer)	P	33.7	Y (0.145)	12
44	Liu 2007 [74] a	China	Asian	39/50	NINCDS/ADRDA and DSM-IV	PCR (original primer)	P	57.0	Y (0.311)	7
45	Han 2008 [75] a	China	Asian	55/59	NINCDS/ADRDA	PCR (original primer)	P	61.1	Y (0.441)	7
46	Vardy 2009 [38]	UK	North European	94/188	NINCDS/ADRDA	PCR (insertion specific primer)	H	47.9	Y (0.980)	10
47	Miners 2009 [76]	UK	North European	86/49	CERAD	PCR (insertion specific primer)	A	34.7	Y (0.487)	8
48	Ning 2010 [77]	China	Asian	138/469	NINCDS/ADRDA and DSM-III-R	PCR (original primer)	P	61.3	Y (0.528)	11
49	Nirmal 2011 [78]	India	Asian	95/130	DSM-IV	PCR (insertion specific primer)	P	45.4	Y (0.937)	10
50	Cousin 2011 [79]	France	South Caucasian	421/460	NINCDS/ADRDA	PCR (original primer)	P	44.5	Y (0.582)	10
51	Lucatelli 2011 [80]	Brazil	South American	35/67	NINCDS/ADRDA and DSM-IV	PCR (original primer)	P	53.7	Y (0.072)	8
52	Yang 2011 [81]	China	Asian	257/137	NINCDS/ADRDA	PCR (insertion specific primer)	P	74.1	Y (0.213)	12
53	Zhang 2014 [82] a	China	Asian	96/102	NINCDS/ADRDA and DSM-IV	PCR (original primer)	H	49.5	Y (0.999)	8


NINCDA/ADRDA: The National Institute of Neurological and Communicative Disorders and Stroke-Alzheimer’s Disease and Related Disorders Association; DSM: Diagnostic and Statistical Manual of Mental Disorders; CERAD: Consortium to Establish a Registry for Alzheimer’s Disease; P: population-based controls; M: Mixed*;* A: autopsy controls; H: Hospital-based controls; HWE: Hardy-Weinberg equilibrium; Y: Yes; N: No.

a Chinese-language studies.

### Pooled prevalence of I/D polymorphism in controls

Overall, the eligible studies included 8153 cases and 14932 controls, and all these samples were genotyped. The pooled frequencies of the *ACE* I allele in control populations demonstrated variation among geographic location/ethnicity groups. Frequencies of the I allele were highest among Asians (59.3%, 95%CI = 54.5–63.9%, using random-effect model), followed by North Europeans (46.9%, 95%CI = 46.2–47.7%, using fixed-effect model) and South Caucasians (39.7%, 95%CI = 36.6–42.8%, using random-effect model). Sensitivity analyses excluding the HWE-deviated studies showed similar results.

### Meta-analysis results

Summaries of the odds ratios for different comparisons were provided in Table 2. In brief, the associations between I/D polymorphism and SAD risk were revealed using allelic comparison (OR = 1.09. 95%CI = 1.01–1.17), homozygote comparison (OR = 1.17, 95%CI = 1.01–1.34) and the dominant model (OR = 1.16, 95%CI = 1.04–1.29). However, FPRP values at the pre-specified prior probability of 0.001were all higher than 0.2 (Table 3), indicating that the associations were not noteworthy. Otherwise, the recessive model showed no significant association in the overall comparisons and all subgroup analyses.

**Table 2:** Summary odds ratio and heterogeneity of the I/D polymorphism on SAD risk.

Variables	N	Allelic comparison (I vs D)			Additive model (II vs DD)			Additive model (ID vs DD)			Recessive model (II vs ID+DD)			Dominant model (II+ID vs DD)		
		OR (95%CI)^a^	*I^2^ (%)*	*P (Q)*	OR (95%CI)^a^	*I^2^ (%)*	*P (Q)*	OR (95%CI)^a^	*I^2^* (%)	*P (Q)*	OR (95%CI)^a^	*I^2^ (%)*	*P (Q)*	OR (95%CI)^a^	*I^2^ (%)*	*P (Q)*
Overall	53	**1.09 (1.01, 1.17)**	**60**	**<10^−3^**	**1.17 (1.01, 1.34)**	**52**	**<10^−3^**	1.11 (0.98, 1.25)	60	<10^−3^	1.08 (0.96, 1.21)	53	<10^−3^	**1.16 (1.04, 1.29)**	**51**	**<10^−3^**
All in HWE	45	**1.11 (1.03, 1.20)**	**56**	**<10** ^−**3**^	**1.19 (1.02, 1.38)**	**49**	**<10** ^−**3**^	1.08 (0.95, 1.24)	58	<10^−3^	1.10 (0.97, 1.25)	52	<10^−3^	**1.17 (1.05, 1.31)**	**45**	**0.001**
English-language articles	48	1.07 (0.99, 1.15)	56	<10^−3^	1.13 (0.98, 1.29)	48	<10^−3^	1.09 (0.96, 1.24)	62	<10^−3^	1.05 (0.94, 1.17)	48	<10^−3^	**1.14 (1.02, 1.27)**	**50**	**<10** ^−**3**^
Geographic location																
North European	18	**1.11 (1.01, 1.21)**	**36**	**0.067**	**1.24 (1.05, 1.48)**	**24**	**0.176**	1.16 (0.91, 1.48)	34	0.100	1.12 (0.97, 1.29)	25	0.165	**1.22 (1.03, 1.45)**	**25**	**0.125**
South Caucasian	14	0.99 (0.91, 1.09)	15	0.292	0.96 (0.80, 1.16)	9	0.356	0.98 (0.83, 1.15)	32	0.118	0.92 (0.78, 1.10)	13	0.307	1.04 (0.93, 1.17)	0	0.525
Asian	15	1.19 (0.96, 1.49)	80	<10^−3^	1.38 (0.94, 2.04)	75	<10^−3^	1.16 (0.86, 1.57)	56	0.004	1.27 (0.96, 1.67)	76	<10^−3^	1.26 (0.91, 1.75)	68	<10^−3^
Other ethnicities	6	1.04 (0.82, 1.31)	25	0.150	1.02 (0.69, 1.52)	39	0.148	1.24 (0.90, 1.73)	41	0.131	0.90 (0.69, 1.18)	0	0.421	1.15 (0.82, 1.63)	54	0.053
Study quality																
High (>9)	28	1.05 (0.97, 1.15)	63	<10^−3^	1.10 (0.94, 1.30)	54	<10^−3^	1.02 (0.88, 1.25)	65	<10^−3^	1.05 (0.91, 1.21)	63	<10^−3^	1.10 (0.98, 1.23)	45	0.005
Low (≤9)	25	1.14 (0.99, 1.30)	57	<10^−3^	1.27 (0.99, 1.63)	50	0.003	**1.25 (1.02, 1.54)**	**46**	**0.007**	1.13 (0.94, 1.36)	35	0.046	**1.25 (1.01, 1.55)**	**56**	**<10** ^−**3**^
Sample size																
Large (≥250)	28	1.04 (0.95, 1.14)	66	<10^−3^	1.09 (0.92, 1.29)	59	<10^−3^	1.01 (0.87, 1.18)	67	<10^−3^	1.04 (0.90, 1.20)	63	<10^−3^	1.08 (0.96, 1.23)	54	<10^−3^
Small (<250)	25	**1.17 (1.03, 1.32)**	**49**	**<10** ^−**3**^	**1.32 (1.04, 1.66)**	**39**	**0.024**	**1.28 (1.06, 1.55)**	**35**	**0.042**	1.16 (0.96, 1.40)	34	0.048	**1.29 (1.06, 1.56)**	**45**	**0.009**
Source of control																
PB	40	1.08 (0.99, 1.18)	63	<10^−3^	1.15 (0.96, 1.37)	60	<10^−3^	0.98 (0.90, 1.06)	65	<10^−3^	1.08 (0.94, 1.24)	58	<10^−3^	1.13 (0.99, 1.28)	55	<10^−3^
Non PB	13	1.11 (0.95, 1.28)	52	0.015	1.18 (0.98, 1.43)	5	0.400	**1.29 (1.11, 1.50)**	**0**	**0.624**	1.04 (0.86, 1.25)	27	0.175	**1.24 (1.01, 1.52)**	**35**	**0.102**
Date of publication time																
After or during 2003	28	1.06 (0.95, 1.18)	65	<10^−3^	1.10 (0.91, 1.33)	53	0.001	0.94 (0.80, 1.10)	52	0.001	1.10 (0.93, 1.30)	61	<10^−3^	1.05 (0.92, 1.20)	38	0.023
Before 2003	25	**1.12 (1.01, 1.24)**	**56**	**<10** ^−**3**^	**1.24 (1.01, 1.53)**	**52**	**0.001**	**1.30 (1.10, 1.54)**	**54**	**0.001**	1.06 (0.91, 1.23)	41	0.017	**1.28 (1.09, 1.52)**	**58**	**<10** ^−**3**^
Genotyping method																
PCR (insertion-specific)	27	1.06 (0.96, 1.17)	63	<10^−3^	1.14 (0.94, 1.38)	58	<10^−3^	1.11 (0.92, 1.34)	72	<10^−3^	1.03 (0.88, 1.19)	54	0.001	**1.17 (1.01, 1.35)**	**58**	**<10** ^−**3**^
PCR (original primer)	26	1.11 (0.99, 1.25)	59	<10^−3^	1.20 (0.98, 1.47)	47	0.005	1.10 (0.94, 1.28)	33	0.054	1.14 (0.96, 1.36)	51	0.001	1.14 (0.97, 1.33)	44	0.008

N: number of studies*;* OR: odd ratio; CI: confidence interval; I^2^: value of *I^2^* for heterogeneity test; P (Q): p value of the Cochran’s *Q* test for heterogeneity.

a A random-effect model was used when *P (Q)* <0.10 or *I^2^*>50%; otherwise, a fixed-effect model was used.

Bold values are significant associations before the FPRP analyses.

**Table 3:** False-positive report probability values for associations between SAD risk and I/D polymorphism.

Significant association	OR (95%CI)	*P* a	Statistical power^b^	Prior probability				
				0.25	0.1	0.01	0.001	0.0001
Overall analyses								
Allelic comparison (I vs D)	1.09 (1.01, 1.17)	0.030	1	**0.049**	**0.133**	0.628	0.945	0.994
Additive model (II vs DD)	1.17 (1.01, 1.34)	0.030	1	**0.065**	**0.173**	0.698	0.959	0.996
Dominant model (II+ID vs DD)	1.16 (1.04, 1.29)	0.008	1	**0.018**	**0.053**	0.379	0.860	0.984
All in HWE								
Allelic comparison (I vs D)	1.11 (1.03, 1.20)	0.009	1	**0.025**	**0.073**	0.463	0.897	0.989
Additive model (II vs DD)	1.19 (1.02, 1.38)	0.027	0.999	**0.060**	**0.161**	0.679	0.955	0.995
Dominant model (II+ID vs DD)	1.17 (1.05, 1.31)	0.005	1	**0.019**	**0.055**	0.391	0.866	0.985
English language article								
Dominant model (II+ID vs DD)	1.14 (1.02, 1.27)	0.018	1	**0.050**	**0.135**	0.633	0.946	0.994
North European subgroup								
Allelic comparison (I vs D)	1.11 (1.01, 1.21)	0.033	1	**0.050**	**0.138**	0.637	0.947	0.994
Additive model (II vs DD)	1.24 (1.05, 1.48)	0.012	0.983	**0.050**	**0.136**	0.634	0.946	0.994
Dominant model (II+ID vs DD)	1.22 (1.03, 1.45)	0.022	0.990	**0.068**	**0.179**	0.706	0.960	0.996
Low study quality subgroup								
Additive model (ID vs DD)	1.25 (1.02, 1.54)	0.034	0.957	**0.102**	0.253	0.789	0.974	0.997
Dominant model (II+ID vs DD)	1.25 (1.01, 1.55)	0.038	0.952	**0.117**	0.284	0.814	0.978	0.998
Small sample size subgroup								
Allelic comparison (I vs D)	1.17 (1.03, 1.32)	0.018	1	**0.031**	**0.088**	0.515	0.915	0.991
Additive model (II vs DD)	1.32 (1.04, 1.66)	0.021	0.863	**0.058**	**0.155**	0.669	0.953	0.995
Additive model (ID vs DD)	1.28 (1.06, 1.55)	0.011	0.948	**0.035**	**0.098**	0.545	0.924	0.992
Dominant model (II+ID vs DD)	1.29 (1.06, 1.56)	0.010	0.940	**0.027**	**0.076**	0.476	0.902	0.989
Non PB subgroup								
Additive model (ID vs DD)	1.29 (1.11, 1.50)	0.001	0.975	**0.003**	**0.009**	**0.087**	0.489	0.906
Dominant model (II+ID vs DD)	1.24 (1.01, 1.52)	0.037	0.967	**0.106**	0.263	0.797	0.975	0.997
Before 2003 subgroup								
Allelic comparison (I vs D)	1.12 (1.01, 1.24)	0.035	1	**0.080**	0.207	0.742	0.967	0.997
Additive model (II vs DD)	1.24 (1.01, 1.53)	0.037	0.962	**0.123**	0.295	0.822	0.979	0.998
Additive model (ID vs DD)	1.30 (1.10, 1.54)	0.002	0.951	**0.008**	**0.022**	0.200	0.716	0.962
Dominant model (II+ID vs DD)	1.28 (1.09, 1.52)	0.003	0.965	**0.015**	**0.043**	0.333	0.835	0.981
PCR (insertion-specific) subgroup								
Dominant model (II+ID vs DD)	1.17 (1.01, 1.35)	0.035	1	**0.086**	0.221	0.757	0.969	0.997

OR: odd ratio; CI: confidence interval.

a p value for significant test; ^b^ Statistical power was calculated using the number of observations in the meta-analysis and the OR and p value in this table.

Bold values indicated that the prior probability <0.2.

**Figure 2:**
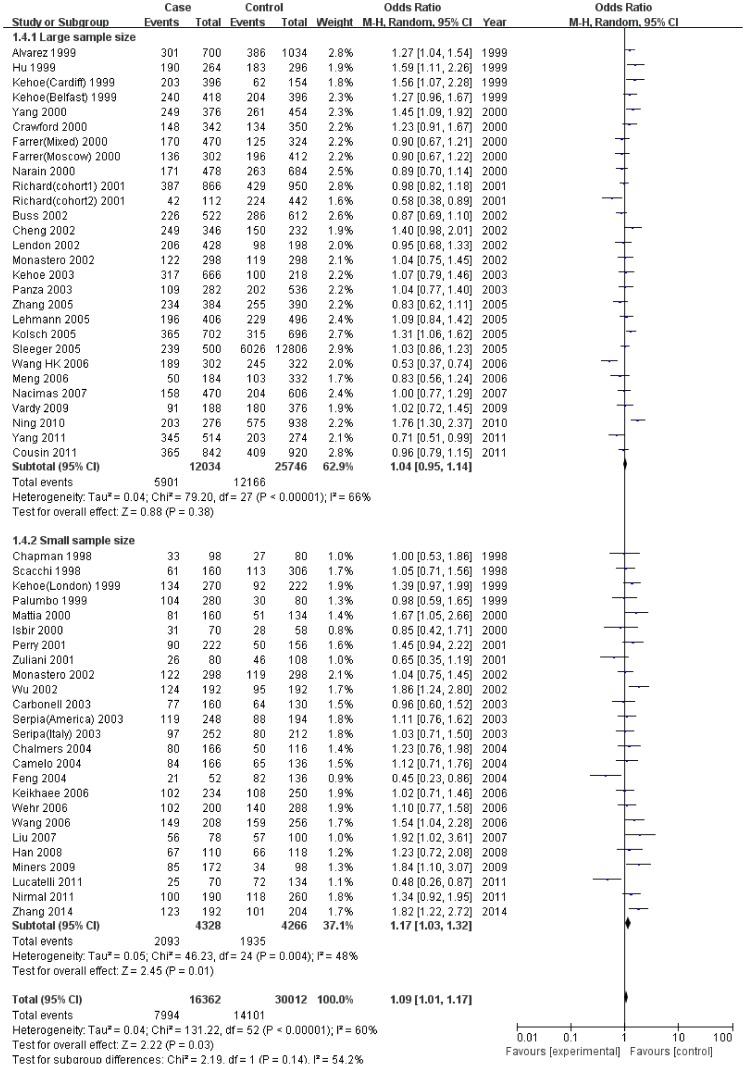
Meta-analysis for the association of SAD risk with *ACE* I/D polymorphism: subgroup analysis by sample size (allelic comparison: I vs D).

**Figure 3:**
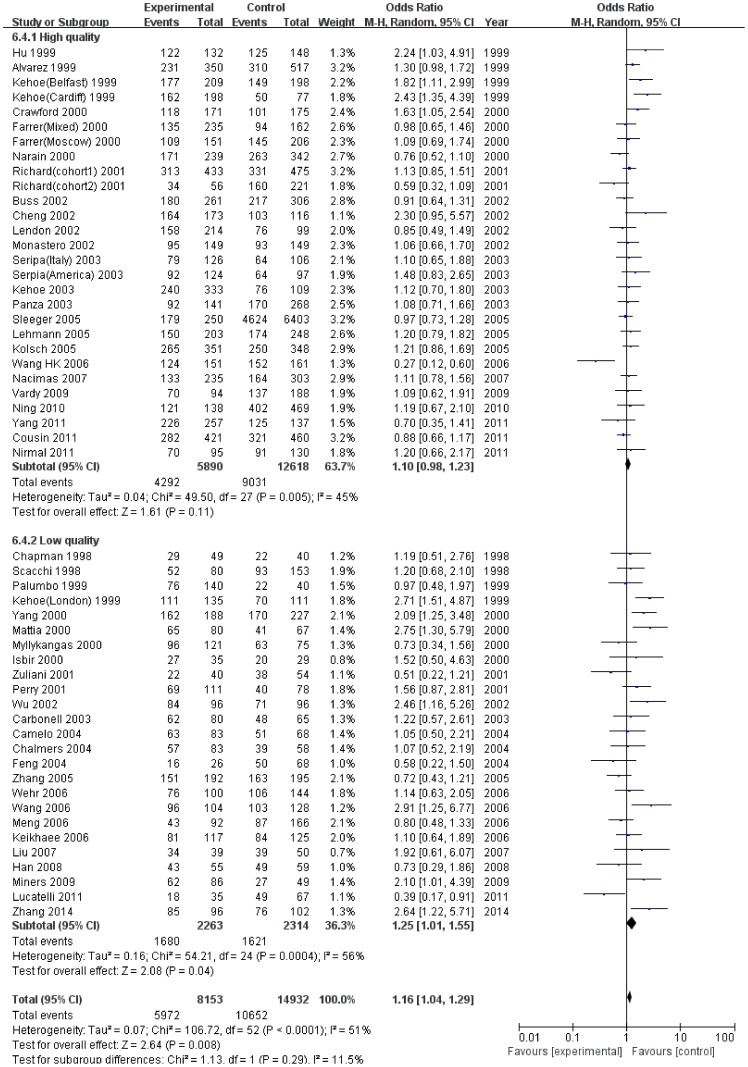
Meta-analysis for the association of SAD risk with *ACE* I/D polymorphism: subgroup analysis by quality appraisal score (dominant model: II+ID vs DD).

When studies were stratified by sample size (Figure 2) and quality appraisal score (Figure 3), the significant associations were especially found in studies with small sample size and the low quality subgroup (Table 2). However, FPRP analyses suggested that the positive results were weak evidence of true associations (Table 3). Otherwise, it was noted that the significant associations between I/D variant and SAD risk disappeared when we restricted to the large sample size studies and the high quality studies (Table 2).

We also investigated potential influence arising from the use of different genotyping method (genotyping with insertion-specific primers prevents mistyping of ID to DD and is thus considered to be more accurate compared with the original method [83]) (Table 2). No statistically significant finding was observed in either the PCR with original primers subgroup or the PCR with insertion-specific primers counterpart, with one exception: a fragile significant finding was seen in the latter subgroup for the dominant model (OR = 1.17, 95%CI = 1.01−1.35), while it was not sufficient robust to withstand the FPRP analysis (Table 3).

When studies were stratified by source of controls (Table 2), significant elevated SAD risks were associated with the I/D polymorphism in the non population-based control subgroup for heterozygote comparison (OR = 1.29, 95%CI = 1.11−1.50) and the dominant model (OR = 1.24, 95%CI = 1.01−1.52), but not among the population-based control subgroup. However, FPRP values of two comparisons were 0.489, 0.975 respectively, indicating the associations were not reliable (Table 3).

The data were additionally stratified by publication time (Table 2). Significant increase associations were found before 2003 using allelic comparison (OR = 1.12, 95%CI = 1.01−1.24), additive model (for II vs DD, OR = 1.24, 95%CI = 1.01−1.53; for ID vs DD, OR = 1.30, 95%CI = 1.10−1.54) and the dominant model (OR = 1.28, 9%CI = 1.09−1.52). However, these associations were not observed between 2004–2014 in all genetic models. The cumulative meta-analysis also illustrated that the exaggerated effect was observed in the earliest study and the accumulated evidence hovered around the conventional 5% significance level until 2002 (Figure S1).

In the subgroup analysis by geographic location (Table 2), significantly increased risks were found among the North Europeans for allelic comparison (OR = 1.11, 95%CI = 1.01−1.21), homozygote comparison (OR = 1.24, 95%CI = 1.05−1.48) and the dominant model (OR = 1.22, 95%CI = 1.03−1.45). However, the associations did not pass the FPRP analyses (Table 3). Otherwise, no associations were detected in South Caucasians, Asians and mixed population groups in any genetic models.

### Sensitivity analyses

Sensitivity analyses showed that no single study notably changed the pooled ORs, indicating that the results of this meta-analysis were stable. Furthermore, after exclusion of HWE-deviated studies, the corresponding pooled ORs did not change significantly (Table 2). However, when we restricted to the English-language studies, only the dominant model between I/D variant and SAD risk remained significant (Table 2), suggesting that a potential language bias was possible. This significant association did not pass the FPRP analysis either (Table 3).

### Heterogeneity analyses

Moderate heterogeneity existed in the overall comparisons (Table 2). When we analyzed data by subgroups, heterogeneity was decreased only in several groups, including North European and South Caucasian populations, studies with other ethnicities, studies with non population-based controls. However, heterogeneity was remained in other subgroups (Table 2). Among all the covariates investigated by meta-regression analyses, sample size (P = 0.023) and publication time (P = 0.009) were factors that contributed to the observed heterogeneity across all studies under the heterozygote comparison (Table S2 in File S1). However, combining with these two factors could only explained 40.48% of the τ^2^ value in heterozygote comparison, indicating that sample size and publication time could explain part of the heterogeneity, but notable heterogeneity still existed. Otherwise, HWE, language, geographic location, quality of studies, sample size, publication time, source of controls and genotyping methods did not contribute the heterogeneity across the overall studies under other genetic comparisons (P>0.05) (Table S2 in File S1). Galbraith plots spotted at least fourteen studies (studies were spotted as the outliers in at least two genetic models) [11], [45], [47], [50], [56], [64], [65], [71], [72], [77], [80]–[82] as the outliers and the possible major sources of heterogeneity in the analyses of total studies (Figure S2a–e). It was noted that 9 [45], [50], [56], [64], [71], [72], [77], [81], [82] of these 14 studies belonged to Asian subgroup. However, we did not try to reduce the obvious heterogeneity by excluding these fourteen studies because it might be unacceptable and could cause some biases by excluding too many studies [84].

### Publication bias

**Figure 4:**
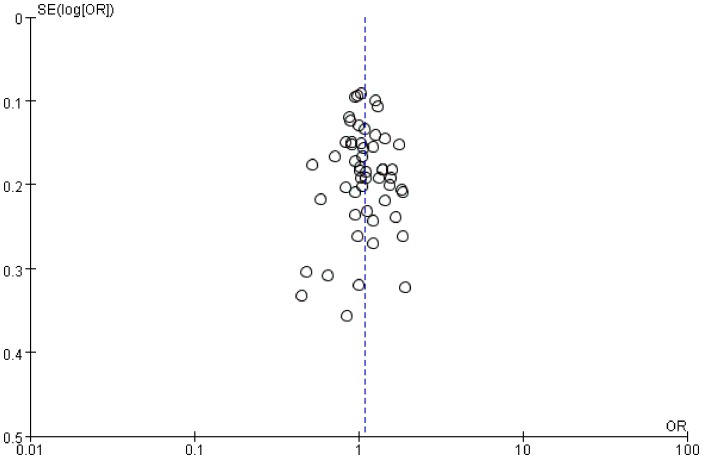
Funnel plot for the association of SAD risk with *ACE* I/D polymorphism (allelic comparison: I vs D).

Funnel plots, Begg's and Egger's tests were performed to assess the publication bias. Funnel plots did not reveal obvious evidence of asymmetry (Figure 4), and all the p values of Begg's tests and Egger's tests were greater than 0.05 (Table S3 in File S1), providing statistical evidence of the funnel plot's symmetry. Ultimately, the results did not suggest any evidence of publication bias.

## Discussion

Previous studies investigating the association between I/D polymorphism and SAD risk have provided controversial results, and most of these studies involved relatively small samples, which were difficult to assess any genetic effects reliably. Meta-analysis has been recognized as an important tool to more precisely define the effect of selected genetic polymorphism on the risk for complex disease [85]. A meta-analysis in 2005 showed that D homozygote was at reduced of AD risk [14]. However, the previous meta-analyses did not cover any studies published in Chinese, which could lead to selection bias and might bias the effect estimate of a meta-analysis. Furthermore, since 2005, sixteen new articles have been published. Hence, to provided the most comprehensive assessment of the association between I/D polymorphism and SAD risk, an updated meta-analysis of all available studies was performed. And our meta-analysis indicated that the *ACE* I/D polymorphism is unlikely to be a major determining factor in the development of SAD. We believed that our results have made much more powerful and detailed analyses to support our results. First, more studies were included in our meta-analysis. Second, more comprehensive subgroup analyses were conducted, and no significant associations were found when we restricted to the high quality subgroup, the large sample size subgroup and the population-based controls subgroup. Finally, to avoid false positive findings, the FPRP analyses were performed for all significant findings observed in our analyses. And none of these significant associations passed the FPRP analyses, indicating that these associations were weak.

Too many reports of associations between genetic variants and complex diseases were false positive [86]. Many false-positive results were likely to be published due to the widely used significance threshold of p<0.05. Therefore, this meta-analysis adopted FPRP analyses, which is based not only on the observed p value but also on both the statistical power and prior probability of the hypothesis, making our results more reliable [26].

Moderate heterogeneity between studies was identified for all genetic models in the overall comparisons. Common reasons of heterogeneity may include differences in the studied populations (e.g., geographic location), or in sample selection (e.g., source of controls, HWE), or in methods (e.g., genotyping methods), or it may be due to interaction with other risk factors (e.g., sample size, study quality and publication time). The meta-regression analyses indicated that the potential sources of heterogeneity for heterozygote comparison were sample size and publication time. However, the sources of heterogeneity for the other models were not found, suggesting that heterogeneity might also be explained by other confounding factors. Nevertheless, when studies were stratified by geographic location, the heterogeneity was higher in the Asian subgroup while it was decreased in other populations. It was the same with the results of Galbraith plot analyses, which spotted at least 14 studies as the outliers and 9 of these 14 studies belonged to the Asian subgroup. These two analyses provided evidence that a combination of heterogeneous studies from the Asian subgroup contributed to the moderate heterogeneity of overall analyses. However, in the Asian subgroup, meta-regression did not find any sources of heterogeneity (data not shown), suggesting that the heterogeneity in the Asian subgroup might be explained by other confounding factors. In general, more rigorous and uniform studies were required.

In the present study, results from populations with different genetic backgrounds were not the same. The combinations of the South Caucasians studies and the Asian studies showed no significant results. However, results from the North European subgroup were distinct and the pooled *ACE* I allele frequency of the controls showed a modest difference across ethnicities (North European studies: 46.9%; South Caucasian studies: 39.7%; Asian studies: 59.3%). These inconsistent data may be explained by the different genetic background across ethnicities. Nevertheless, owing to the greater FPRP values of significant associations in the North European subgroup, the observed ethnic difference in this meta-analysis was also likely to be caused by chance because continued reliance on the standard p value criterion of 0.05 to define statistical significance without consideration of power or prior probability may generated a fluctuated risk estimate [26]. Thus, large and carefully designed studies on ethnicity difference were also needed to provide the best evidence for these possible associations.

When studies were stratified by sample size, significant elevated SAD risks were associated with the I/D polymorphism in the small sample size subgroup. However, small sample with limited participants was often accompanied with selection biases, and lacked sufficient power to support or deny an association [87]. It was therefore speculated that the small sample size subgroup might overestimate the magnitude of association between I/D variant and SAD risk. Moreover, these significant associations in the small sample size subgroup were weak as they did not pass the FPRP analyses. Finally, when we restricted to the large sample size subgroup, no statistically significant finding was observed in any genetic models.

In the stratification analysis by source of controls, significant associations were observed using heterozygote comparison and the dominant model in the non populations-based subgroup. However, the genotype distributions in the non population-based studies may not be representative of the general population. Given the fact that these associations were not noteworthy (did not pass the FPRP analyses) and no significant association was found in the population-based studies, we thought that this significant association for the I/D variant might be a spurious finding. More and larger population-based studies were required to further clarify the association between I/D variant and SAD risk.

Despite our efforts in performing a deeper analysis, some limitations also exist in our meta-analysis. First, in most overall and subgroup analyses, moderate heterogeneity was detected and might have potential impact in the pooled results. Due to the limited information, subgroup analyses and meta regression according to other confounding factors were not performed. Second, sensitivity analysis limited to English-language studies suggested that a potential language bias was possible. However, we only included studies published in Chinese and English, more studies published in other languages should be concerned. Finally, lack of individual participants' data has restricted further adjustments by other covariables, such as *APOE ε4* status, gender, etc.

In conclusion, given that all significant associations could not pass the FPRP analyses and no significant association was detected in the high quality studies, the large sample size studies and the population-based studies, we suggest that the *ACE* I/D polymorphism is unlikely to be a major determining factor in the development of SAD.

## Supporting Information

Figure S1 **Cumulative meta-analysis of the relation between** ***ACE*** **I/D polymorphism and risk of SAD (I vs D).** Each study was used as an information step. The vertical dotted line is the summary odds ratio. Bars represent 95% confidence interval (CIs)
(TIF)

Figure S2 **Galbraith plots of association between I/D polymorphism and SAD risk.** Each number represents a separate study for the indicated association and the number is the number of the respective study included into the meta-analysis (shown in Table 1). (a) allelic comparison, I vs D; (b) additive model, II vs DD; (c) additive model, ID vs DD; (d) recessive model, II vs ID+DD; (e) dominant model, II+ID vs DD.
(DOC)

File S1 (1) Table S1 Scale used for quality assessment of studies of the association between *ACE* I/D polymorphism and SAD risk. (2) Table S2 The univariate meta-regression results of the association of the I/D polymorphism and risk of SAD. (3) Table S3 Results of Begg’s tests and Egger’s tests for overall analyses.
(DOC)

Checklist S1 **PRISMA Checklist for systematic review and meta-analysis.**
(DOC)

Checklist S2 **Genetic association studies checklist.**
(DOCX)
