# Supplementary figures and images for: Angiotensin-Converting Enzyme Insertion/Deletion Polymorphism Is Not a Major Determining Factor in the Development of Sporadic Alzheimer Disease: Evidence from an Updated Meta-Analysis
Source: PLoS One. 2014 Oct 31;9(10):e111406. doi: 10.1371/journal.pone.0111406 (PMC4216072; doi:10.1371/journal.pone.0111406)

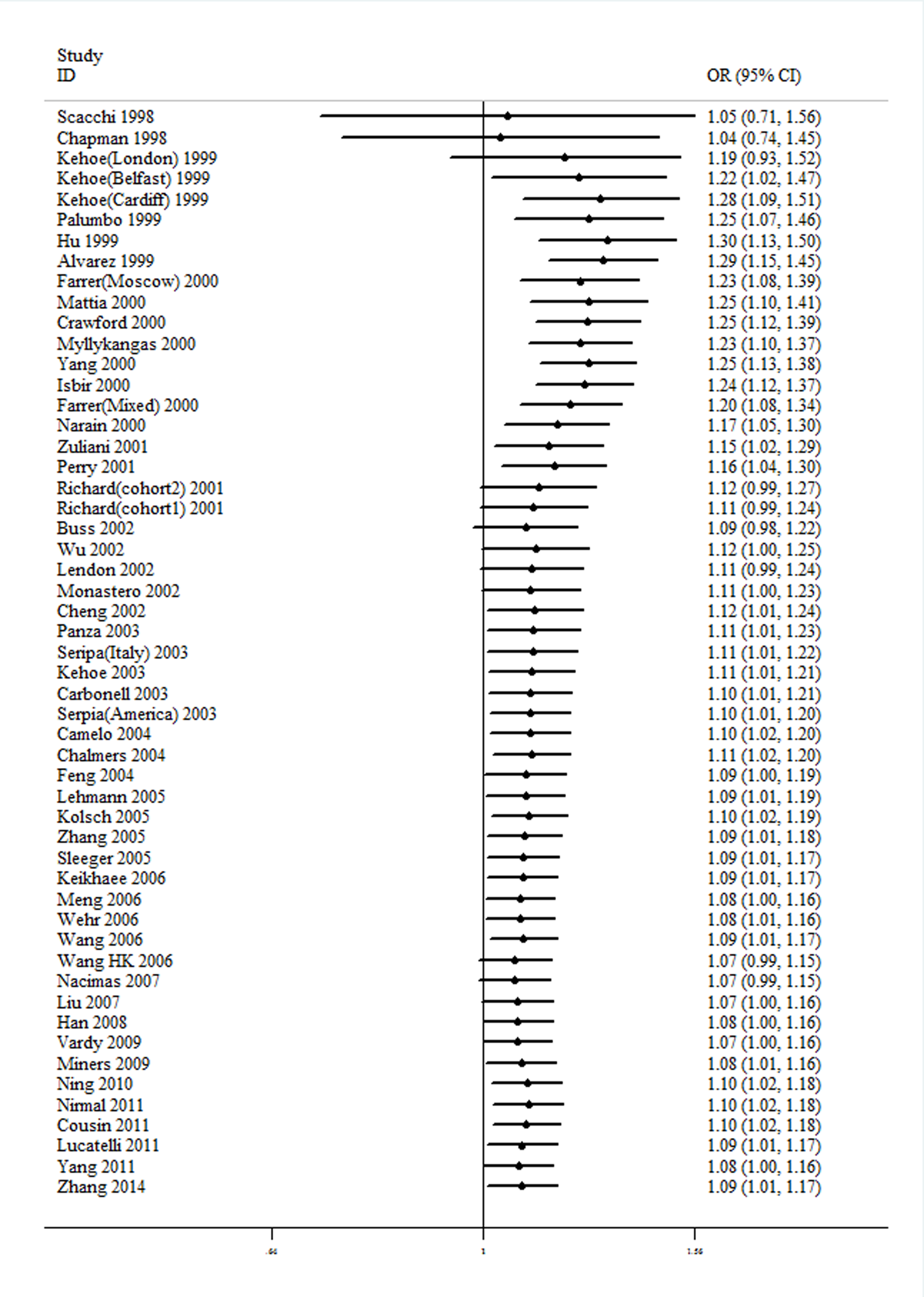

Supplement: Figure S1 — Cumulative meta-analysis of the relation between ACE I/D polymorphism and risk of SAD (I vs D). Each study was used as an information step. The vertical dotted line is the summary odds ratio. Bars represent 95% confidence interval (CIs) (TIF) [file pone.0111406.s001.tif]
