# Supplementary material for: Angiotensin-Converting Enzyme Insertion/Deletion Polymorphism Is Not a Major Determining Factor in the Development of Sporadic Alzheimer Disease: Evidence from an Updated Meta-Analysis
Source: PLoS One. 2014 Oct 31;9(10):e111406. doi: 10.1371/journal.pone.0111406 (PMC4216072; doi:10.1371/journal.pone.0111406)
Supplement: Figure S2 — Galbraith plots of association between I/D polymorphism and SAD risk. Each number represents a separate study for the indicated association and the number is the number of the respective study included into the meta-analysis (shown in Table 1). (a) allelic comparison, I vs D; (b) additive model, II vs DD; (c) additive model, ID vs DD; (d) recessive model, II vs ID+DD; (e) dominant model, II+ID vs DD. (DOC) [file pone.0111406.s002.doc]

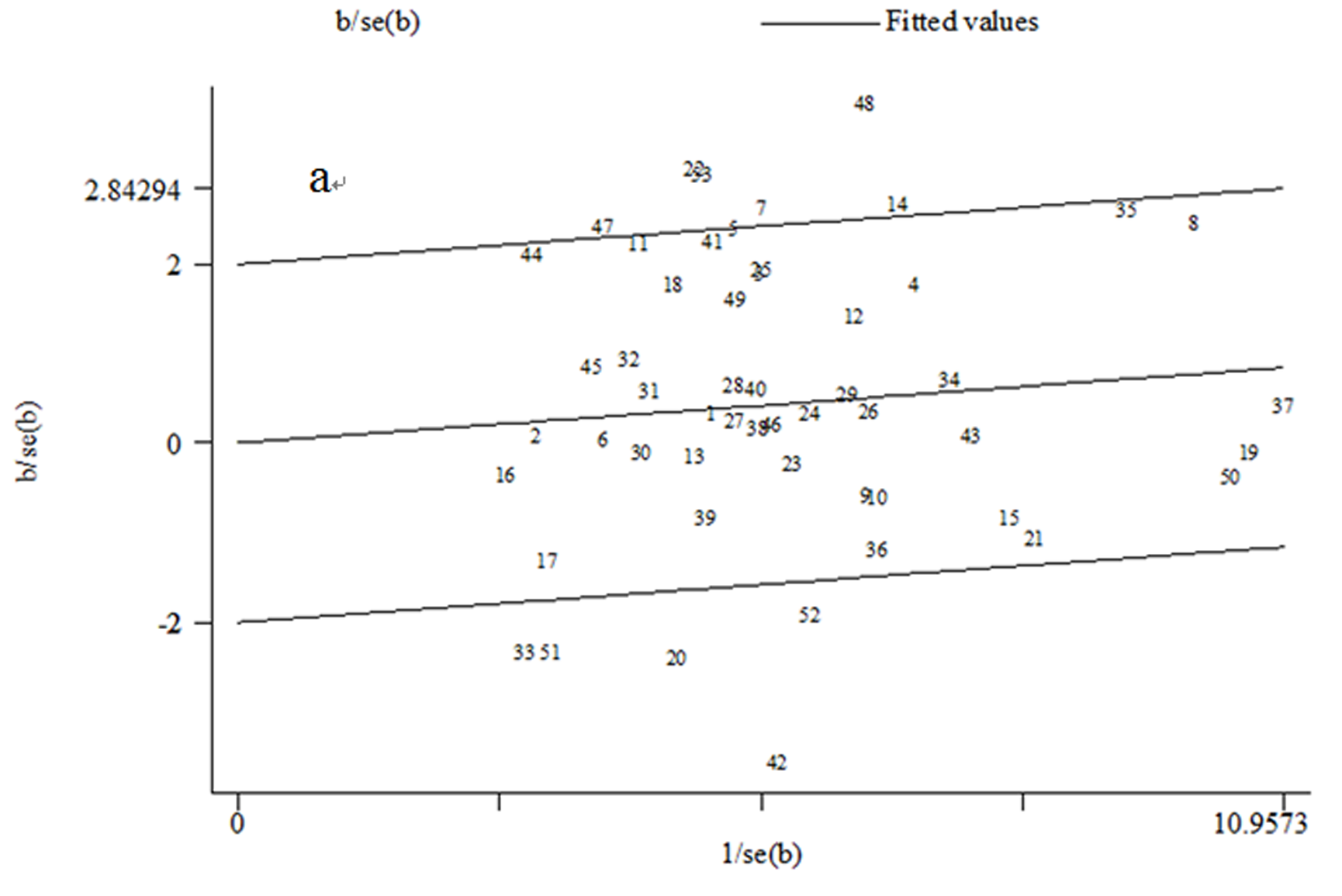


Figure S2a. Galbraith plots of association between I/D polymorphism and SAD risk. Each number represents a separate study for the indicated association and the number is the number of the respective study included into the meta-analysis (shown in Table 1). (a) allelic comparison, I vs D.


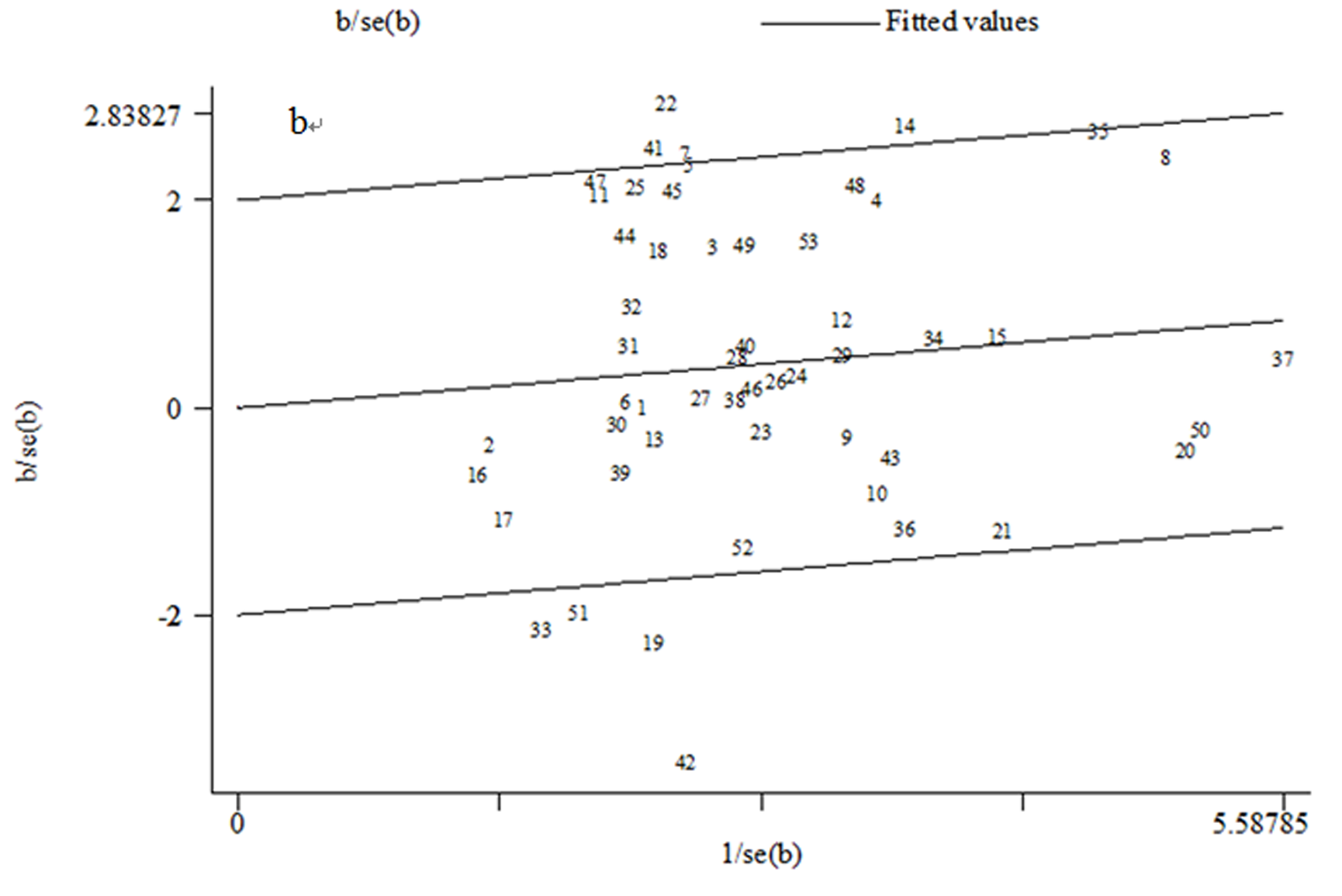


Figure S2b. Galbraith plots of association between I/D polymorphism and SAD risk. Each number represents a separate study for the indicated association and the number is the number of the respective study included into the meta-analysis (shown in Table 1). (a) additive comparison, II vs DD.


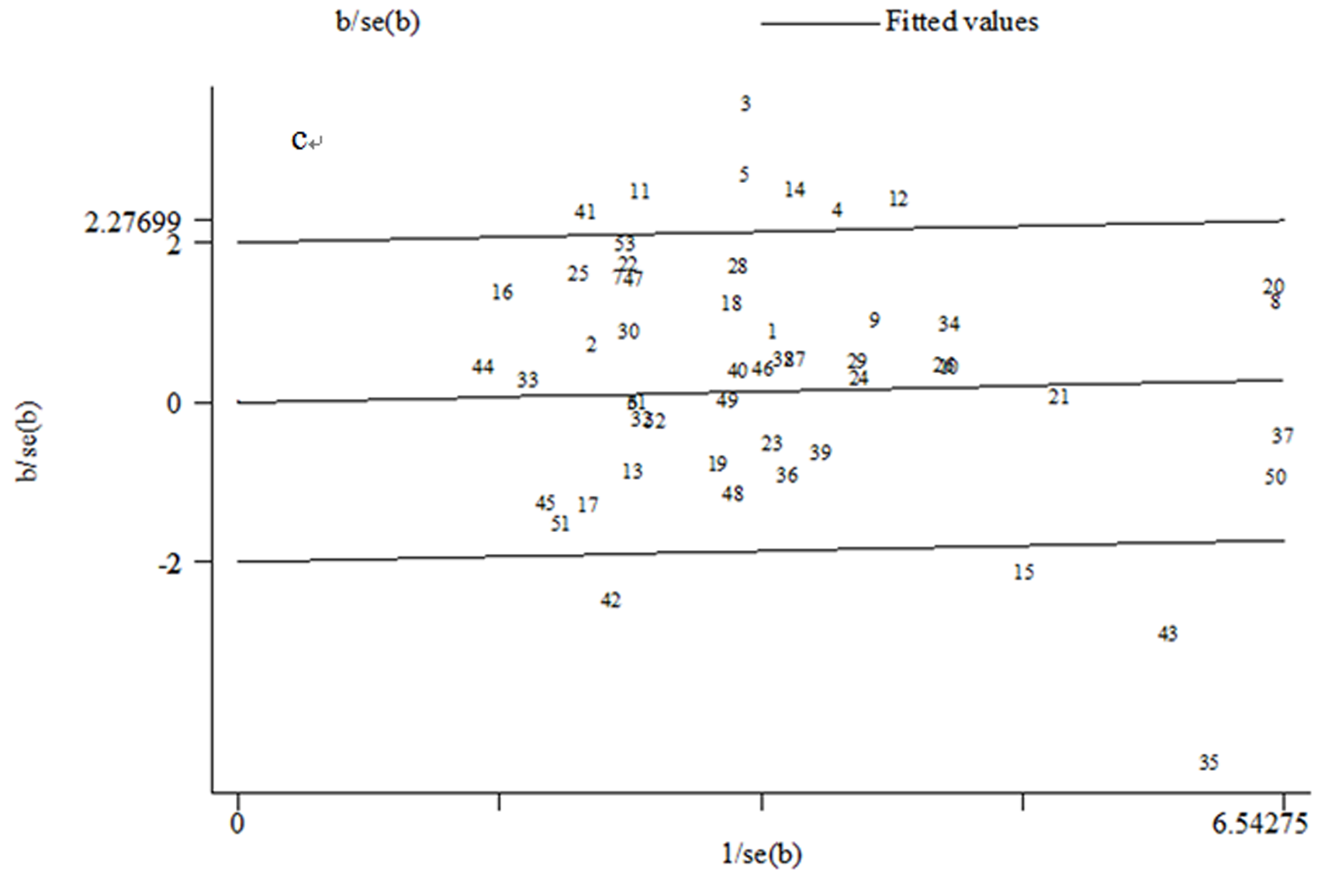


Figure S2c. Galbraith plots of association between I/D polymorphism and SAD risk. Each number represents a separate study for the indicated association and the number is the number of the respective study included into the meta-analysis (shown in Table 1). (a) additive comparison, ID vs DD.


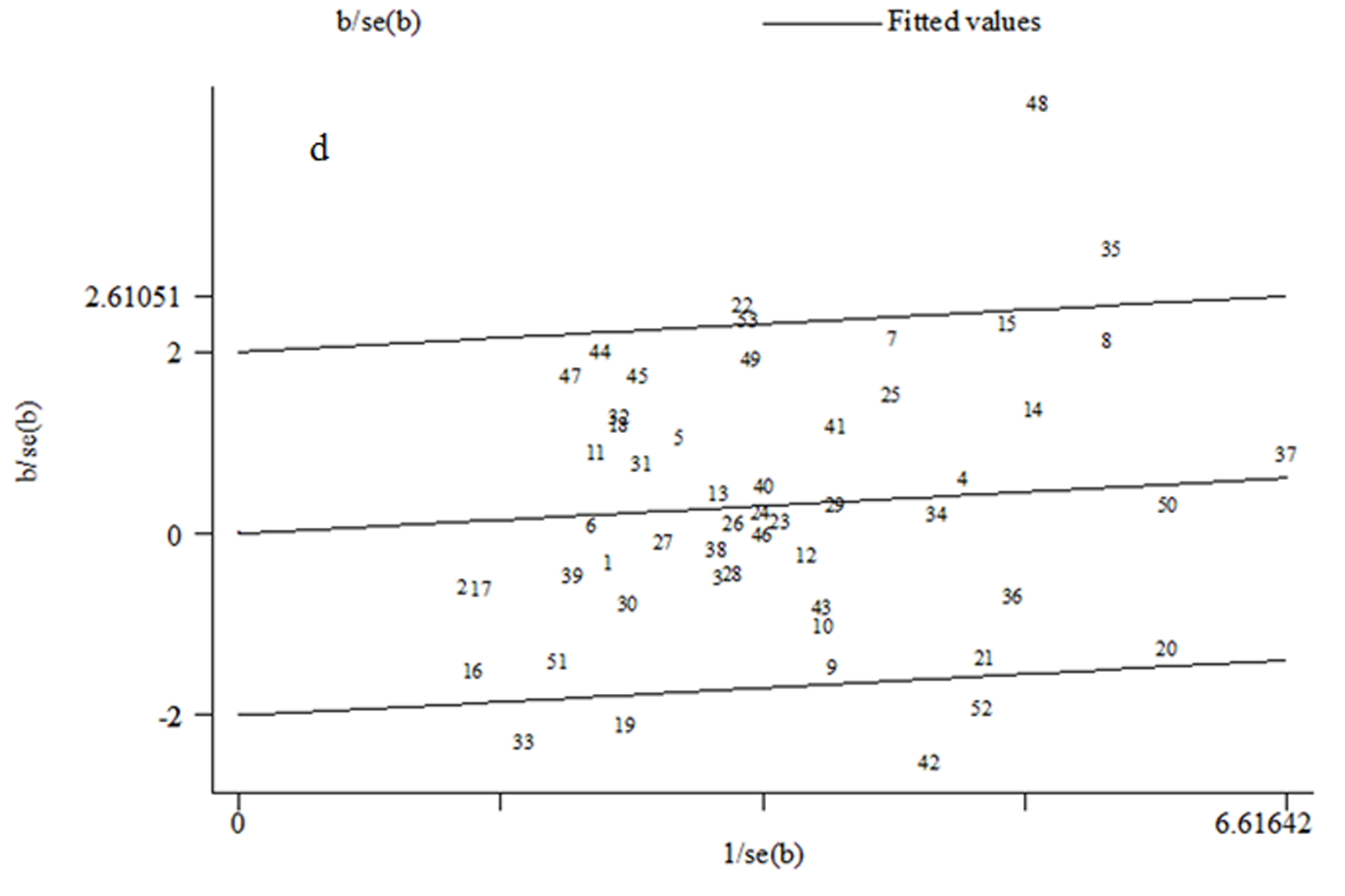


Figure S2d. Galbraith plots of association between I/D polymorphism and SAD risk. Each number represents a separate study for the indicated association and the number is the number of the respective study included into the meta-analysis (shown in Table 1). (a) recessive model, II vs ID + DD..


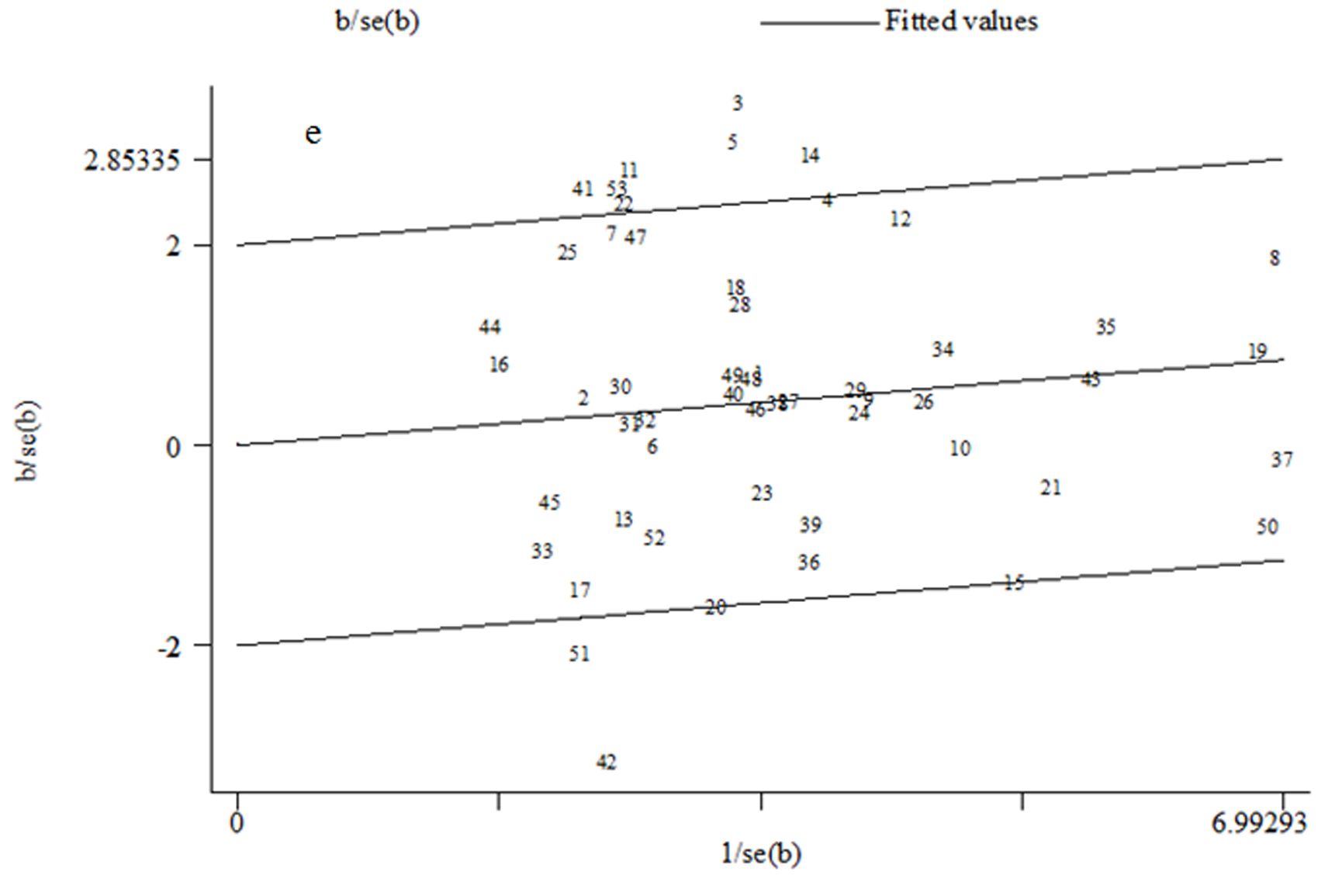


Figure S2e. Galbraith plots of association between I/D polymorphism and SAD risk. Each number represents a separate study for the indicated association and the number is the number of the respective study included into the meta-analysis (shown in Table 1). (a)dominant model, II + ID vs DD.
