# Supplementary material for: Angiotensin-Converting Enzyme Insertion/Deletion Polymorphism Is Not a Major Determining Factor in the Development of Sporadic Alzheimer Disease: Evidence from an Updated Meta-Analysis
Source: PLoS One. 2014 Oct 31;9(10):e111406. doi: 10.1371/journal.pone.0111406 (PMC4216072; doi:10.1371/journal.pone.0111406)
Supplement: File S1 — (1) Table S1 Scale used for quality assessment of studies of the association between ACE I/D polymorphism and SAD risk. (2) Table S2 The univariate meta-regression results of the association of the I/D polymorphism and risk of SAD. (3) Table S3 Results of Begg’s tests and Egger’s tests for overall analyses. (DOC) [file pone.0111406.s003.doc]

**Supplementary Table**

**Negative association between Angiotensin-converting enzyme gene insertion/deletion polymorphism and sporadic Alzheimer disease: evidence from an updated meta-analysis**

Xue-Bin Wang^1^, Ning-Hua Cui^2^, Jie Yang^1^, Xue-Ping Qiu^1^, Jia-jia Gao^1^, Na Yang^1^, Fang Zheng^*1^

^1^Center for Gene Diagnosis, Zhongnan Hospital of Wuhan University, Wuhan 430071, Hubei, China

^2^ Department of Clinical Laboratory, Children's Hospital of Zhengzhou, Zhengzhou 450053, Henan, China

^*^Corresponding author: Fang Zheng

Email address: zhengfang@whu.edu.cn

Telephone number: 862767813233

Fax number: 862767813497

| **Table S1.** Scale used for quality assessment of studies of the association between *ACE* I/D polymorphism and SAD risk. | |
| --- | --- |
| Criteria | Score |
| **Representativeness of cases** |  |
| Consecutive/randomly selected from case population with clearly defined sampling frame | 2 |
| Consecutive/randomly selected from case population without clearly defined sampling frame or with extensive inclusion/exclusion criteria | 1 |
| No method of selection described | 0 |
| **Source of controls** |  |
| Population/neighbor based | 2 |
| Non Population based (Hospital/Autopsy/Mixed based) | 1 |
| Not described | 0 |
| **Hardy-Weinberg equilibrium in controls** |  |
| Hardy-Weinberg equilibrium | 1 |
| Hardy-Weinberg disequilibrium | 0 |
| **Genotyping method** |  |
| PCR with insertion-specific primers | 2 |
| PCR with original primers | 1 |
| Not describe | 0 |
| **Genotyping blinding** |  |
| Blinded | 1 |
| Unblinded/Not mention | 0 |
| **Association assessment** |  |
| Association assessment between genotypes and SAD with appropriate statistics and adjustment for confounders | 2 |
| Association assessment between genotypes and SAD without appropriate statistics and adjustment for confounders | 1 |
| Inappropriate statistic used | 0 |
| **Total sample size** |  |
| ≥250 | 2 |
| ≥150but <250 | 1 |
| <150 | 0 |

| **Table S2.** The [univariate](javascript:void(0);) meta-regression results of the association of the I/D polymorphism and risk of SAD. | | | | | | | |
| --- | --- | --- | --- | --- | --- | --- | --- |
| Comparison | Covariates | Coefficient | Standard error | P value | 95% confidence interval | τ^2^ value (%) | I^2^ res (%) |
| Allelic comparison |  |  |  |  |  |  |  |
|  | HWE | -0.139 | 0.113 | 0.223 | -0.366～0.088 | -0.73 | 60.47 |
|  | Language | 0.271 | 0.163 | 0.103 | -0.056～0.599 | 10.33 | 58.83 |
|  | Geographic location | 0.024 | 0.034 | 0.483 | -0.044～0.093 | -1.11 | 60.66 |
|  | Study quality | -0.081 | 0.085 | 0.344 | -0.252～0.090 | 1.62 | 60.23 |
|  | Sample size | -0.114 | 0.846 | 0.182 | -0.284～0.055 | 5.93 | 59.50 |
|  | Source of control | 0.024 | 0.098 | 0.804 | -0.172～0.221 | -3.92 | 61.15 |
|  | Date of publication | -0.052 | 0.833 | 0.533 | -0.220～0.115 | -2.41 | 60.96 |
|  | Genotyping method | -0.047 | 0.084 | 0.580 | -0.215～0.121 | -2.60 | 61.04 |
| Homozygote comparison |  |  |  |  |  |  |  |
|  | HWE | -0.099 | 0.201 | 0.624 | -0.503～0.305 | -4.70 | 52.74 |
|  | Language | 0.419 | 0.281 | 0.142 | -0.145～0.983 | 10.55 | 50.78 |
|  | Geographic location | 0.033 | 0.063 | 0.608 | -0.094～0.159 | -3.36 | 52.84 |
|  | Study quality | -0.150 | 0.157 | 0.346 | -0.465～0.166 | 2.49 | 52.03 |
|  | Sample size | -0.194 | 0.157 | 0.223 | -0.509～0.121 | 6.11 | 51.46 |
|  | Source of control | 0.052 | 0.176 | 0.771 | -0.303～0.406 | -5.71 | 53.13 |
|  | Date of publication | -0.121 | 0.153 | 0.433 | -0.429～0.187 | -2.86 | 52.71 |
|  | Genotyping method | -0.052 | 0.154 | 0.738 | -0.362～0.258 | -4.87 | 53.11 |
| Heterozygote comparison |  |  |  |  |  |  |  |
|  | HWE | 0.119 | 0.173 | 0.495 | -0.229～0.467 | -2.67 | 60.15 |
|  | Language | 0.193 | 0.280 | 0.497 | -0.370～0.753 | 0.14 | 60.22 |
|  | Geographic location | 0.015 | 0.056 | 0.783 | -0.097～0.128 | -2.43 | 60.05 |
|  | Study quality | -0.200 | 0.131 | 0.133 | -0.463～0.063 | 7.25 | 58.10 |
|  | **Sample size** | **-0.285** | **0.121** | **0.023** | **-0.528～-0.042** | **15.30** | **57.31** |
|  | Source of control | 0.224 | 0.146 | 0.130 | -0.069～0.517 | 7.58 | 57.48 |
|  | **Date of publication** | **-0.326** | **0.120** | **0.009** | **-0.568～-0.084** | **22.70** | **52.94** |
|  | Genotyping method | -0.001 | 0.131 | 0.998 | -0.263～0.262 | -3.77 | 60.62 |
| Recessive model |  |  |  |  |  |  |  |
|  | HWE | -0.128 | 0.151 | 0.403 | -0.432～0.176 | -2.76 | 52.98 |
|  | Language | 0.449 | 0.221 | 0.057 | -0.011～0.892 | 12.69 | 50.27 |
|  | Geographic location | 0.044 | 0.047 | 0.349 | -0.050～0.137 | -1.27 | 53.16 |
|  | Study quality | -0.071 | 0.122 | 0.565 | -0.312～0.175 | -2.05 | 53.46 |
|  | Sample size | -0.107 | 0.123 | 0.389 | -0.354～0.140 | -0.05 | 53.08 |
|  | Source of control | -0.031 | 0.136 | 0.820 | -0.305～0.243 | -3.05 | 53.22 |
|  | Date of publication | 0.044 | 0.118 | 0.709 | -0.193～0.281 | -4.11 | 53.59 |
|  | Genotyping method | -0.107 | 0.117 | 0.364 | -0.343～0.128 | 0.84 | 52.68 |
| Dominant model |  |  |  |  |  |  |  |
|  | HWE | -0.095 | 0.166 | 0.568 | -0.428～0.238 | -4.32 | 51.86 |
|  | Language | 0.268 | 0.267 | 0.321 | -0.269～0.804 | 3.95 | 51.13 |
|  | Geographic location | 0.009 | 0.053 | 0.859 | -0.097～0.115 | -5.27 | 52.02 |
|  | Study quality | -0.131 | 0.125 | 0.296 | -0.382～0.119 | 3.36 | 50.82 |
|  | Sample size | -0.175 | 0.124 | 0.163 | -0.423～0.073 | 10.67 | 49.80 |
|  | Source of control | 0.093 | 0.142 | 0.517 | -0.193～0.379 | -2.39 | 51.54 |
|  | Date of publication | -0.194 | 0.118 | 0.106 | -0.430～0.042 | 7.81 | 49.66 |
|  | Genotyping method | 0.029 | 0.123 | 0.815 | -0.217～0.275 | -5.65 | 52.02 |
| Bold values indicated that these covariates were the sources of heterogeneity (P value <0.05) | | | | | | | |

| **Table S3**. Results of Begg’s tests and Egger’s tests for overall analyses. | | |
| --- | --- | --- |
| Comparison | P (Begg) | P (Egger) |
| Allelic comparison (I vs D) | 0.586 | 0.861 |
| Additive model (II vs DD) | 0.945 | 0.879 |
| Additive model (ID vs DD) | 0.448 | 0.107 |
| Recessive model (II vs ID + DD) | 0.279 | 0.241 |
| Dominant model (II + ID vs DD) | 0.421 | 0.316 |
| P (Begg): the p value for Begg’s tests; P (Egger): the p value for Egger’s tests. | | |
