# Supplementary material for: Angiotensin-Converting Enzyme Insertion/Deletion Polymorphism Is Not a Major Determining Factor in the Development of Sporadic Alzheimer Disease: Evidence from an Updated Meta-Analysis
Source: PLoS One. 2014 Oct 31;9(10):e111406. doi: 10.1371/journal.pone.0111406 (PMC4216072; doi:10.1371/journal.pone.0111406)
Supplement: Checklist S2 — Genetic association studies checklist. (DOCX) [file pone.0111406.s005.docx]

**Meta-analysis on Genetic Association Studies Checklist | PLOS ONE**

|  | Item | Section name and paragraph number within manuscript |
| --- | --- | --- |
|  | **Introduction** |  |
| 1 | Provide a detailed justification for the polymorphism studied; if a single polymorphism was analyzed, give details as to why others were not included in the meta-analysis. | Introduction (Paragraph 1-2, Page 3-4 |
| 2 | Provide a detailed justification for the population(s) and clinical condition studied. | Introduction (Paragraph 2, Page 3-4) |
|  | **Methods** |  |
| 3 | Provide full details of the search strategy employed; outline the full electronic search strategy –specific combination of keywords and any limits applied- for at least one database. Specify whether synonyms of polymorphisms/genes (e.g. SNP number) were searched. | Literature search (Materials and methods, Page 4) |
| 4 | Report full details on the inclusion and exclusion criteria applied for selecting studies.  *Please list the excluded articles and the reasons for exclusion of each article in a supplementary file.* | Inclusion criteria (Materials and methods, Page 4)) |
| 5 | Provide details on how the quality of the studies included in the analyses was assessed. | Quality score assessment (Materials and methods, Page 5)/Table S1 |
| 6 | Describe steps taken to contact study authors to identify additional studies and to request missing data. | Data extraction (Materials and methods, Page 5) |
| 7 | Describe how environmental effects were adjusted for, if this adjustment was not conducted, outline the reasons for this. | This adjustment was ont conducted becaused of raw data |
| 8 | Describe the methods of handling heterogeneity/between-study variance. | Statistical analysis (Materials and methods, Page 6) |
| 9 | Describe how the Hardy-Weinberg equilibrium and linkage disequilibrium were assessed. | Statistical analysis (Materials and methods, Page 5) |
| 10 | Describe and justify the choice of model for the analyses (per-allele vs per-genotype vs genetic model-free, random effects vs fixed effects). | Statistical analysis (Materials and methods, Page 5-6) |
| 11 | Describe whether a sensitivity analysis has been completed. | Statistical analysis (Materials and methods, Page 6) |
| 12 | Describe whether an assessment of the effects of population stratification has been conducted. | Statistical analysis (Materials and methods, Page 6) |
| 13 | Describe whether study-specific results have been assessed and if so the reasons for this (e.g. forest plot). | Statistical analysis (Materials and methods, Page 5-6) |
|  | **Results** |  |
| 14 | Include flow diagram for the studies included in the meta-analysis as the first figure for the manuscript | Study characteristics (Result, Page 7)/Figure 1 |
| 15 | Report details on allele/genotype prevalence. | Pooled prevalence of I/D polymorphism in controls (Result, Page 8)/Table 1 |
| 16 | Report the effect size estimates and p values for each analysis. | Meta-analysis results (Result, Page 8-10)/Table 2/Figure 1,2 |
|  | **Discussion** |  |
| 17 | Discuss the limitations of the meta-analysis, including genotyping errors/bias and publication bias. | The penultimate paragraph (Page 14) |
| 18 | If the meta-analysis identifies an association within a subgroup of the population studied but not another, discuss the implications of these results, and if applicable the possibility of subgroup-specific publication bias. | Paragraph 4-6, Page 13-14 |
| 19 | Discuss the suitability of the sample size employed to the research question and the power of the study. | Paragraph 1 |
